# Immunopathological Roles of Cytokines, Chemokines, Signaling Molecules, and Pattern-Recognition Receptors in Systemic Lupus Erythematosus

**DOI:** 10.1155/2012/715190

**Published:** 2012-01-23

**Authors:** Shui-Lian Yu, Woon-Pang Kuan, Chun-Kwok Wong, Edmund K. Li, Lai-Shan Tam

**Affiliations:** ^1^Department of Medicine and Therapeutics, Prince of Wales Hospital, The Chinese University of Hong Kong, 30-32 Ngan Shing Street, Shatin, Hong Kong; ^2^Department of Rheumatology, Hospital Selayang, Lebuhraya Selayang-Kepong, 68100 Batu Caves, Malaysia; ^3^Department of Chemical Pathology, Prince of Wales Hospital, The Chinese University of Hong Kong, 30-32 Ngan Shing Street, Shatin, Hong Kong

## Abstract

Systemic lupus erythematosus (SLE) is an autoimmune disease with unknown etiology affecting more than one million individuals each year. It is characterized by B- and T-cell hyperactivity and by defects in the clearance of apoptotic cells and immune complexes. Understanding the complex process involved and the interaction between various cytokines, chemokines, signaling molecules, and pattern-recognition receptors (PRRs) in the immune pathways will provide valuable information on the development of novel therapeutic targets for treating SLE. In this paper, we review the immunopathological roles of novel cytokines, chemokines, signaling molecules, PRRs, and their interactions in immunoregulatory networks and suggest how their disturbances may implicate pathological conditions in SLE.

## 1. Introduction

Systemic lupus erythematosus (SLE) is a prototypic systemic autoimmune disease which is characterized by a loss of tolerance to nuclear antigens and various immunological abnormalities, including dysregulated activation of both T and B lymphocytes and subsequent polyclonal activation of circulating B lymphocytes which produces a large quantity of autoreactive antibodies and the formation of immune complexes causing tissue and organ damage [1]. This is a complex process involved interaction between various cytokines, chemokines, signaling molecules, and pattern-recognition receptors (PRRs) in the immune pathways. With the advent of new and advanced technique which include intracellular cytokine analysis by flow cytometry combined with multiplex quantization of cytokine levels in recent years, it had provided us a reasonable understanding of the activation profile of cytokine production and new insight in the immune and cellular mechanism in the pathogenesis of SLE, which further clarify the significance of the current body of literatures. This had provided valuable information on the development of novel therapeutic targets for treating SLE. This article will focus on the recent advances of cytokines, chemokines, signaling molecules, and the role of PRRs in immunopathogenesis in SLE.

## 2. Imbalance of Th1/Th2 Cytokines in SLE

**Figure 1:**
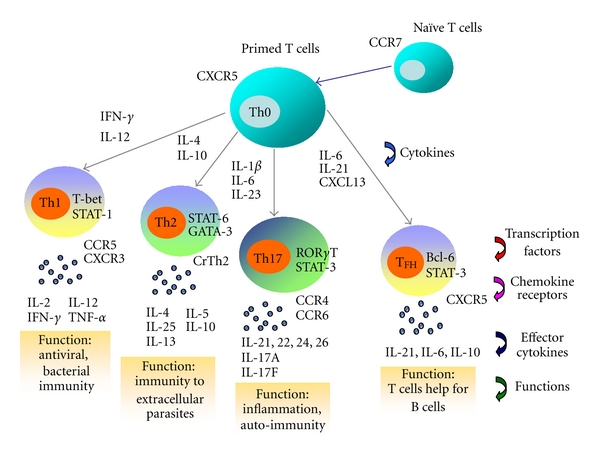
Effector T-cell differentiation (Th1, Th2, Th17 and T_FH_), the expression of transcription factors, effector cytokines, chemokine receptors, and T-cell functions.

Cytokines are a group of small peptides or glycoprotein produced by a wide variety of cells with molecular weights between 8 and 30 kDa. They had been shown to play an essential role in modulating the immune response against foreign or self-antigens. These mediators have been classified according to their cellular source and effector functions, with the paradigmatic T helper (Th)1 and Th2 cytokine families best illustrating this division of function. Th1 cells arise in response to dendritic cells- (DCs-) derived interleukin- (IL-) 12, produce tumor necrosis factor- (TNF-) *α*, interferon- (IFN-) *γ*, and are involved in mediating strong inflammatory responses to intracellular pathogens. IL-4-mediated Th2 cell differentiation results in cells that produce cytokines, including IL-4, IL-5, and IL-13, which mediate antibody responses to extracellular pathogens (Figure 1).

The ratios of Th1 and Th2 cytokines have been investigated to determine the cytokine homeostasis in order to determine whether Th1 or Th2 predominance during the development of SLE [2, 3]. SLE was thought to be a Th2-polarized disease because of the production of auto-antibodies specific for self-antigens [4]. However, significantly elevated cytokines for Th1 response including IL-12, TNF-*α*, and IFN-*γ* were also found in the plasma of SLE patients [5–8]. Th1 dominant immune responses have been generally considered to be pathological in autoimmune disease via the induction of inflammatory reaction. Recently, few cytokines which had been shown to be of great importance in pathogenesis of SLE had surfaced with advent of new technology in detection, which enhances our understanding of their role in SLE-related immune pathway. These cytokines, including IL-12, IL-23, IL-18, IL-21, and IL-33, will be discussed below.

### 2.1. IL-12

IL-12 is a heterodimeric cytokine of 70 kDa comprising covalently linked p40 and p35 subunit which has been shown to be a central stimulator of Th1-related proinflammatory cytokine that induces IFN-*γ* in both innate and adaptive immunity [9, 10]. IL-12 had been suggested to be associated with progression of severe glomerulonephritis [11]. Moreover, mRNA levels of p19, p40, and p35 of IL-12 were found to be significantly higher in active SLE patients compared with those patients with inactive disease [12]. Accordingly, serum level of IL-12 was also found to be significantly elevated in SLE patients, and it is associated with the increased level of Th1 cytokine IFN-*γ* but decreased level of Th2 cytokine IL-13 [5, 13, 14]. Conversely, another study reported the decreased *ex vivo* production of IL-12 from peripheral blood polymorphonuclear leukocytes (PMN) stimulated by lipopolysaccharide (LPS) in patients with active SLE [15] using a different ELISA kit. Recently, the elevated plasma IL-12 concentration has been shown to exhibit positive correlation with systemic lupus erythematosus disease activity index (SLEDAI) in SLE patients with renal impairment, supporting IL-12 could play a pathological role in the development of autoinflammatory response in SLE patients with severe disease, probably through the recruitment of the effector leukocytes to the inflamed tissue for orchestrating the immunoresponse at the site of inflammation [16].

### 2.2. IL-23

IL-23 is a novel heterodimeric cytokine composed of a unique p19 subunit, and a common p40 subunit shared with IL-12. IL-23 shares similar intracellular signal transduction molecules with IL-12, therefore both cytokines exhibit some overlapping function in promoting cellular immunity [17]. Different from IL-12, IL-23 does not promote the development of IFN-*γ*-producing Th1 cells, but is crucial for the expansion of a pathogenic CD4^+^ T-cell population characterized by the production of IL-17 and IL-22 [18, 19]. Recent studies had shown that the mRNA levels of IL-23p19 were significantly higher in active SLE patients when patients were stratified into different disease activity groups, thereby suggesting that IL-23 should play a role in SLE disease exacerbation [12]. Moreover, the likely significance of IL-23 in autoinflammatory responses was further supported by a more recent report indicated that Th1 transcription factor T-bet could upregulate IL-23 receptor expression and the differentiation of Th1 and Th17 cells in autoimmunity [20] (Figure 1). IL-23 has been reported to enhance the IL-17 secretion by peripheral blood mononuclear cells (PBMC) from healthy subjects [20]. Moreover, the pathogenic Th17 subgroup expresses elevated level of IL-23 receptor via the activation by T-bet, thereby representing a distinct inflammatory Th cell lineage for the development of organ-specific autoimmune inflammation [18, 20–22]. In order to better elucidate the involvement of IL-23 in the IL-23/IL-17 autoinflammatory axis and the immunopathological mechanisms of the activation of Th17 cells in SLE, Wong et al. have used IL-23 as an activating agent to demonstrate the direct involvement of IL-23 in the IL-23/IL17 inflammatory axis. It acts to induce a distinct T-cell activation state that produces IL-17 as the effector cytokine that promotes the autoinflammatory responses in SLE [16].

### 2.3. IL-18

IL-18 was originally identified as a factor that enhances IFN-*γ* production in macrophages, T lymphocytes, and DCs [23]. Previous studies also reported that the involvement of this Th1-related cytokine in initiating both innate and acquired immune responses [24, 25]. It has been elucidated that IL-18 along with IL-12 is a potent inducer of the inflammatory mediators by T lymphocytes, causing severe inflammatory disorders in autoimmune diseases such as rheumatoid arthritis (RA) [26]. In SLE, previous studies by our group and others have demonstrated the increased levels of IL-18 in serum/plasma of affected persons, which positively correlated with disease severity [13, 27–29]. Of interest is the elevated urinary IL-18 levels that were found significantly increased in patients with established acute tubular necrosis [30] and the increases within 24 hours after kidney transplantation in patients with delayed allograft dysfunction [31], suggesting that IL-18 may serve as an prognostic marker of renal involvement useful to identify patients at risk of renal failure. Possible pathogenic role of IL-18 in lupus has been studied in a mouse model of progressive disease, demonstrating that IL-18 has a multifaceted role in autoimmune lupus, being apparently involved both in the effector phases of the late organ damage and, in some organs, in the initial pathogenic events [32, 33].

### 2.4. IL-21

IL-21 is a pleiotropic cytokine, produced by CD4^+^ Th cells, that modulates the differentiation and function of T cells, B cells, natural killer (NK) cells, and DCs by binding to the receptor composing of the IL-21 receptor-*α* (IL-21R*α*) and the common *γ* chain [34, 35]. Recent study has intimated that IL-21 can mediate the differentiation and generation of follicular helper T cells (T_FH_) [34, 36] (Figure 1). Nevertheless, autocrine production of IL-21 from T_FH_ cells can potently stimulate the differentiation of B cells into antibody-forming cells through IL-21R [37]. As a result, dysregulation of T_FH_ cell function may relate to the pathogenesis of SLE. IL-21 has been shown to contribute to the development of autoimmune diseases in different animal models of SLE, experimental autoimmune encephalomyelitis, and RA [35]. The genetic association of IL-21 polymorphisms has also been demonstrated in SLE [38]. Recent animal study has revealed that elevated production of IL-21, T_FH_ dysfunction within germinal centers, and aberrant positive selection of germinal center B cells are required for the production of autoantibodies and systemic autoimmunity [39, 40].

### 2.5. IL-33

IL-33, a novel member of the IL-1 cytokine family [41], has recently been shown to be involved in the pathogenesis of chronic inflammatory disease [42–44] similar to other family members IL-1 and IL-18 [45]. IL-33 is responsible for the protection against helminth infections and prevention of atherosclerosis by promoting Th2 immune responses [46]. The IL-33 receptor, consisting of ST2 and IL-1 receptor accessory protein, is also widely expressed, particularly on Th2 cells and mast cells [47], to mediate important effector Th2 functions [48]. Although the elevated ST2 protein in the sera of SLE and other patients with autoimmune diseases has been reported [49], its causal relationship with disease activity is still unclear. Recently, significantly elevated serum soluble ST2 (sST2) but not IL-33 has been detected in SLE patients, and the levels of sST2 were found to correlate with the disease activity and severity of these medical conditions. It therefore suggested that the sST2 level may have a potential role as a surrogate marker of disease activity [44]. In this study, no correlation was found between serum IL-33 level and sST2 level, lupus disease activity, or specific organ involvement. In contrast, others reported that serum IL-33 level was significantly increased in SLE compared with healthy controls (HCs). Increased IL-33 level was significantly associated with thrombocytopenia, erythrocytopenia, and anti-SSB antibody, suggesting IL-33 may exert biologic effects on erythrocytes and platelets or their precursors in SLE [43]. In summary, the role of the IL-33/ST2 system in the pathogenesis of SLE remained unclear.

## 3. Imbalance of Th1/Th2 Transcription Factors in SLE

Although the control of the Th1/Th2 imbalance has been unclear, there is growing evidence to suggest that two major transcription factors, T-box expressed in T cells (T-bet) and GATA binding protein 3 (GATA-3), are the determining factors of T-helper cell differentiation [50]. T-bet, a Th1-specific transcription factor, has been postulated to initiate Th1 development while inhibiting Th2 cell differentiation [51]. GATA-3 is a member of the GATA zinc finger protein family, and enhances the development of the Th2 phenotype while inhibiting Th1 cells [52–54]. Recent study had demonstrated that the mRNA levels of T-bet and IFN-*γ* and the relative expression levels of T-bet/GATA-3 and IFN-*γ*/IL-4 were significantly higher, in contrast to the lower expressions of GATA-3 and IL-4, in SLE patients [55]. There were also significant correlations in mRNA expression of T-bet with IFN-*γ* and of GATA-3 with IL-4. Additionally, the relative expressions of T-bet/GATA-3 and IFN-*γ*/IL-4 were found to correlate with lupus disease activity. Moreover, the elevated plasma Th1/Th2 cytokine ratio of IL-18/IL-4 was also shown to correlate positively with disease activity in all SLE patients, suggesting the functional activation of peripheral blood Th1 cells in SLE patients. Thus, previous study provided us with new insight that ratio of T-bet/GATA-3 expression is more informative than the level of either transcription factor alone, which may be disproportionately affected by the changes in their coexpression in cell populations. The T-bet/GATA-3 expression ratio not only enhances our understanding of Th1/Th2 polarization, it may also serve as a supplementary tool for further assessment of Th1/Th2 status and development of SLE disease activity (Figure 1).

## 4. Th17-Mediated Inflammation of SLE

Apart from Th1 and Th2 cells, there is a novel subset of IL-17 producing effector T helper cells, called Th17 cells, whose dysregulation is thought to participate in the pathogenesis of SLE [56, 57]. Transforming growth factor (TGF)-*β*, IL-6, IL-21, and IL-23 have been implicated for Th17 formation [58, 59]. Other proteins involved in their differentiation are signal transducer and activator of transcription 3 (STAT3) and the retinoic-acid-receptor-related orphan receptors alpha (ROR*α*) and gamma (ROR*γ*) [58]. Moreover, effector cytokines associated with this cell type are IL-17, IL-21, and IL-22 [60] (Figure 1). We herein highlighted some of the biological effects of IL-17 implication for Th17-mediated inflammation of SLE.

 IL-17 is a type I 17-kDa transmembrane protein that comprises six members and five receptors mostly produced by activated T cells [61]. It is a pleiotropic proinflammatory cytokine that enhances T-cell priming and stimulates epithelial, endothelial, and fibroblastic cells to produce multiple proinflammatory mediators, including IL-1, IL-6, TNF-*α*, and chemokines [62]. Additionally, IL-17 also exerts its effects through the recruitment of monocytes and neutrophils by increasing the local production of chemokines (IL-8, monocyte chemoattractant protein-1, growth-related oncogene protein-*α*), the facilitation of T-cell infiltration and activation by stimulating the expression of intercellular adhesion molecule-1 by T cells as well as the amplification of the immune response by inducing the production of IL-6, prostaglandin E_2_, granulocyte-macrophage colony-stimulating factor, and granulocyte colony-stimulating factor [63]. Lastly, this cytokine synergizes with other cytokines, in particular with IL-1*β*, TNF-*α*, and IFN-*γ* [63].

 Wong et al. have demonstrated that SLE patients have higher plasma/serum levels of IL-17 than HCs [13, 16, 56], which positively associated with SLE disease activity [16]. Accordingly, the frequency of IL-17-producing T cells is increased in peripheral blood of SLE patients [16, 64]. Significant levels of IL-17 and IFN-*γ* were detected in T cells from SLE patients [64]. Additionally, overproduction of total immunoglobulin G (IgG), antidouble stranded DNA, and IL-6 by PBMC of patients with lupus nephritis was observed upon the stimulation with IL-17 [65], suggesting a potential role of IL-17 in human lupus progression. On the other hand, no elevation of IL-17 was found in serum of cohort of Japanese lupus patients [66]. Most recent evidence suggested that the ability of regulatory T cells (T_regs_) to express IFN-*γ* and IL-17 was impaired in SLE patients, whereas the proportion of T_regs_ was similar between SLE patients and HCs [67]. Additionally, studies in mice support the concept that IL-17 and Th17 cells may be involved in the development of lupus nephritis [56, 68]. For instance, IL-17 was recently found to be critical for the formation of autoreactive germinal centres in autoimmune BXD2 mice, a strain that develops a lupus-like syndrome [69]. In a spontaneous mouse model of lupus, the New Zealand Black (NZB) mice, stimulation of splenocytes with nucleosomes as an autoantigen results in the activation of large numbers of IL-17-secreting T cells [70]. Upon adoptive transfer to naïve recipient mice, IL-23-dependent IL-17 producing CD4^+^ effector T-cell subset Th17 can invade the target organ and promote the development of organ-specific autoimmune inflammation. Consistently, Wong et al. also found that the proinflammatory cytokine IL-23 and IL-12 can promote the disease severity by activating pathogenic Th1 and Th17 cells via the induction of downstream Th1 chemokine CXCL10 and inflammatory cytokine IL-17 in SLE, demonstrating that the IL-23/IL-17 axis of inflammation and related molecules may arise as a therapeutic target for treating autoimmune disease.

## 5. Chemokines in SLE

Chemokines in itself refer to a group of smaller cytokines (mass between 8 to 12 kDa) with chemotactic properties, which are classified into four families according to the location of cysteine residues. The four chemokine groups are CC, C, CXC, and CX_3_C, where C is a cysteine and X is any amino acid residue [71]. These small molecules have had well-defined roles in directing cell migration necessary for the initiation of T cell immune response, attraction of appropriate effector cells to sites of inflammation, and regulation of differential recruitment of T helper (Th1 and Th2) lymphocytes [72–74]. There has been growing evidence suggesting that infiltration of T lymphocytes and other leucocytes into the sites of inflammation plays a critical role in organ involvement in SLE [75]. Recent studies have also shown that chemokines and their receptors are intimately involved in regulating organ-specific leucocyte trafficking and inflammation, suggesting their important roles in the pathophysiology of autoimmune diseases such as RA, multiple sclerosis, and SLE [76–78]. Chemokine CXCL13 in emerging studies had consolidated the important role of these chemokines in pathogenesis of SLE. Other chemokines that will be briefly discussed in this article mainly include CC and CXC chemokines which had been shown to play some roles in SLE disease.

### 5.1. CXCL13

CXCL13/B lymphocyte chemoattractant (BLC) is a small cytokine belonging to the CXC chemokine family that is produced by cells in the omentum, peritoneal macrophages, and DCs [79, 80], which is selectively chemotactic for B cells including both the B1 and B2 subsets by interacting with specific chemokine receptor CXCR5 [79, 81]. The accumulation of B1 cells in the peritoneal cavity and spleen are responsible for the body cavity immunity and the production of autoantibody for the development of autoimmune disease in the murine model [79, 82, 83]. Elevated levels of B1 cells have been documented in patients with autoimmune disorders such as Sjogren's syndrome and RA [84, 85]. Previous studies using murine model of SLE showed that CXCL13 is highly produced by CD11b^+^ CD11c^+^ DCs in the target organs including thymus and kidney for the chemoattraction of B1 cells into target organ [83, 86–88]. Therefore, the elevated expression of CXCL13 by myeloid dendritic cells (mDCs) in the target organs may play a crucial role in breaking the immune tolerance in the thymus leading to the activation of self-reactive CD4^+^ Th cells and the recruitment of autoantibody producing B cells in the development of murine lupus [83, 87, 88]. In addition to that, studies have revealed that CXCL13 can induce the trafficking of distinct CXCR5^+^ T cells designated as T_FH_ which are specifically involved in high-affinity IgG production in germinal centers developed within B-cell follicles of secondary lymphoid tissues including lymph nodes, spleen, and tonsils [36, 89–91]. CD4^+^ T_FH_ cells, located at B-cell follicles, provide a T helper function to B cells and represents one of the most numerous and important subsets of effector T cells in lymphoid tissue [37, 92]. Several studies demonstrated that B-cell chemokine CXCL13 is ectopically and highly expressed in thymus and kidney in murine model for SLE. Studies on humans also demonstrated that serum CXCL13 level was significantly elevated in SLE patients and the elevation correlated significantly with SLE disease activity [93, 94]. As anti-TNF-*α* treatment was found to be able to reduce the plasma level of CXCL13 in RA patients [95], it had been postulated that serum level of CXCL 13 can act as a disease activity marker for both RA and SLE patients.

### 5.2. CC Chemokines

Monocyte chemoattractant protein-1 (MCP-1/CCL2) is a prototype CC chemokine, which can attract monocytes, T cells, NK cells, and basophils [96, 97]. An increase of serum MCP-1/CCL2 was observed with the progression of disease activity in SLE patients compared to HCs [98]. Further investigation reported that cerebral spinal fluids (CSF) MCP-1/CCL2 levels were significantly higher in neuropsychiatric syndromes of systemic lupus erythematosus (NPSLE) patients than those non-NPSLE patients [99]. Regulated upon activation, normal T-cell-expressed and secreted (RANTES)/CCL5 is another CC chemokine which attracts monocytes, memory T cells, and NK cells [100]. Increased plasma RANTES/CCL5 concentrations were found in SLE patients more than in controls, and correlated significantly with SLEDAI score [101]. Moreover, the expression of miR-125a was found to contribute to the elevated expression of RANTES/CCL5 in SLE [102]. In addition to that, studies from animal models and patients with lupus nephritis demonstrated that inflammatory chemokines, especially CCL2 and CCL5, are detectable in kidney tissues and urine before other signs of inflammation [103–106]. With this finding, urine chemokines had been proposed as a possibility to serve as biomarkers for renal SLE flare [107], suggesting that the reduced plasma concentration of these circulating chemokines in lupus patients with renal involvement may result from a protein leakage in the urine.

### 5.3. CXC Chemokines

Interferon-gamma inducible protein-10 (IP-10)/CXCL10 and monokine induced by gamma-interferon (MIG)/CXCL9, the prototype of the CXC family, have chemotactic activity mainly for activated Th1 cells and are involved in the pathogenesis of various Th1-dominant autoimmune diseases [71, 108]. Their synthesis and expression from neutrophils, macrophages, and other immune cells are induced by IFN-*γ*, and this response is suppressed by IL-10 and IL-4 [71, 109]. Th1 cells and IFN-*γ* had been shown to be important for cell-mediated inflammation in developing autoimmune disease such as SLE [5], thus implicated that these chemokines might have an important role in pathogenesis of SLE. Furthermore, several studies have shown that levels of IP-10/CXCL10 and MIG/CXCL9 were significantly elevated in active SLE [98, 110, 111]. Moreover, Okamoto et al. reported that IP-10/CXCL10 was upregulated in the central nervous system (CNS) fluid of NPSLE [112, 113], suggesting that IP-10/MCP-1 ratio in CSF is a useful diagnostic marker of NPSLE [112]. On the other hand, CXC chemokines CXCL8 and CXCL1 are potent chemoattractants and activators of T cells, neutrophils, thereby enhancing their proinflammatory and proangiogenic activities [114]. They also stimulate neutrophil degranulation to release reactive oxygen radicals, thereby inducing an acute inflammatory reaction [115, 116]. They had also been shown to be significantly elevated in serum of patient with active lupus, and the elevation was associated with disease activity [111].

## 6. Intracellular Signaling Pathways in SLE

Signal transduction refers to an ordered biochemical process by which a signal or stimulus is transferred within a single cell. This cascade begins with binding of extracellular signaling molecules to cell surface receptors, triggering an initial stimulus that propagated into the cytoplasm. Nowadays, the most well-known and established signal transduction pathway that has been identified is mitogen-activated protein kinase (MAPK) pathway. MAPKs are serine and threonine protein kinases that can be activated by phosphorylation in response to extracellular stimuli, such as mitogens, growth factors, cytokines, and osmotic stress [117, 118]. Nuclear translocation of activated MAPKs can induce and transactivate transcription factors including nuclear factor- (NF-) *κ*B and activator protein 1, which facilitate the modulation of gene transcription in cellular activation, proliferation, apoptosis, and the expression of cytokines, chemokines, adhesion molecules, and metalloproteinases [117, 118]. Three main distinct MAPKs, p42/p44 extracellular signal-regulated kinase (ERK), c-Jun NH2-terminal protein kinase (JNK), and p38 MAPK, have been identified in mammalian cells. The activation of NF-*κ*B, JNK, and p38 MAPK plays crucial roles in cytokine-mediated signaling pathways regulating the release of chemokines and the expression of adhesion molecules of eosinophils and Th cells [119–121]. Activation of p38 MAPK has been shown to be crucial for B-cell activation leading to Ig production, and p38 MAPK regulates the production of a number of cytokines, including IL-6 that promotes the differentiation and survival of plasma cells [122]. Moreover, B-cell-activating factor of the TNF family, an essential factor for B-cell activation and differentiation, was regulated through JNK and p38 MAPK [123]. Furthermore, nuclear factor of activated T cells (NFAT), a downstream transcription factor of the ERK and JNK pathways, is essential for T and B lymphocyte activation and differentiation [124], and specific anti-NFAT drug therapy has been shown to be pharmacologic armamentarium against RA, inflammatory arthropathies, and related autoimmune disorders [125].

## 7. Interaction between Cytokines, Chemokines, and Signaling Molecules in SLE

As mentioned before, immunopathogenesis of SLE is a complex process that involved the interaction and synergistic effect of various cytokines, chemokines, and signaling molecules which perpetuate the disease activity in SLE. This section below will highlight the recent update on the interaction between all these agents in promoting the disease activity in SLE.

### 7.1. Role of IL-18 and Chemokines

The potential role of IL-18 and chemokines in the exacerbation of SLE disease had been highlighted in a study, which provided valuable information on the development of SLE disease markers [111]. In this study, plasma concentration of CXCL10, CCL5, CXCL9, CXCL8, CXCL1, and CCL2 was significantly elevated in SLE patients and the elevation was correlated significantly with disease activity. Furthermore, plasma concentration of IL-18 was found to be correlated positively with production of CXCL10, CXCL9, CXCL1, and CXCL8 in SLE patients, it was also shown to be a potent costimulus for the induction of these chemokine release from activated PBMC as there was a significant increase in *ex vivo* production of these inflammatory chemokines when their PBMC were cultured in the presence of IL-18.

 This enhances our knowledge that successful delivery of the appropriate population of leucocytes to sites of acute inflammation will depend on the repertoire of inducible chemokines synthesized locally, and the temporal expression of chemokine receptors on the leucocytes. Meanwhile, the chemokine expressions are influenced by proinflammatory cytokines, mainly IL-18, to present in the local environment of the cells at the time of stimulation. Furthermore, inflammatory activities of IL-18, together with the induction of Th1 cytokine IFN-*γ* and the activation of Th cells, natural killer cells (NK), and cytotoxic T lymphocytes-inflammatory chemokines, may even enhance the Th1-mediated inflammatory process, the activation of NK and T cells, and the migration of macrophages for initiating and perpetuating the Th1 immune response in SLE. In summary, the correlation of raised plasma concentration and *ex vivo* production of inflammatory chemokines with disease activity, and their association with IL-18, supports that the chemotaxis of Th1/Th2 lymphocytes and neutrophils is important in SLE pathogenesis.

### 7.2. Role of CXCL13 and IL-21

A recent study [93] had shown that CXCL13 and IL-21 may relate with the immunopathogenesis mediated by the function of T_FH_ cells in SLE as serum level of all these cytokines were found to be significantly elevated in lupus patient with the increase in CXCL13 concentration correlated positively and significantly with SLEDAI score. Furthermore, cell surface expression of CXCR5 on Th and B cells and IL-21R on B cells was found to be significantly lower in SLE patients, which indicated that most differentiated T_FH_ cells migrate out from circulation into lymphoid organ upon activation during the disease development of SLE. This piece of information suggests that the elevated production of CXCL13, BAFF, and IL-21 may be associated with the function of T_FH_ for the immunopathogenesis in SLE, and CXCL13 may serve as a potential disease marker of SLE.

### 7.3. Role of IL-23, IL-17, IL-18, Th17, and CXCL10

The pathogenic role of IL-23/IL-17 autoinflammatory axis in SLE had been elucidated in a recent study [16]. First, parallelly elevated plasma IL-12, IL-17, and CXCL10 concentrations exhibited positive correlation with the SLEDAI in their lupus patients with renal impairment, which supported that these cytokines cascade could play a pathological role in the development of autoinflammatory response in SLE patients with severe disease, through the recruitment of the effector leukocytes into the inflamed tissue for orchestrating the immunoresponse at the site of inflammation. Second, when using IL-23 as activator, the CD3 and CD28 costimulated PBMC responded with an aberrant *ex vivo* production of IL-17, which provided robust evidence on the direct involvement of IL-23 in the IL-23/IL-17 inflammatory axis, which acts to induce a distinct T-cell activation state that produces IL-17 as the effector cytokine that promotes the autoinflammatory responses in SLE. Third, *ex vivo* production of IL-12, IL-23, and IL-17 from PMBC was significantly enhanced by the presence of IL-18 which indicated that the expressions of inflammatory cytokines IL-12, IL-23, and IL-17 and activation of Th17 cells are in part influenced by proinflammatory cytokine IL-18 present in the local environment of the cells during stimulation. IL-23-mediated activation of IL-17-producing Th cells in SLE patients may closely be influenced by IL-18 activation, which orchestrates the inflammation of SLE. In conclusion, proinflammatory cytokine IL-18 and IL-12 family cytokines IL-12 and IL-23 can promote the disease severity by activating pathogenic Th1 and Th17 cells via the induction of downstream Th1 chemokine CXCL10 and inflammatory cytokine IL-17 in SLE.

### 7.4. Role of MAPK, IL-18, and CXCL10

As for the roles of MAPK transduction pathway in pathogenesis of SLE, highly abnormal ERK and NF-*κ*B activities in T lymphocytes of lupus patients had been reported [126, 127]. The lyn kinase deficiency in B lymphocytes and decreased ras-MAPK in T lymphocytes had also been demonstrated in SLE patients [128–130]. A recent study had further consolidated the facts that p38 MAPK and JNK are the key signaling molecules in regulating the inflammation-mediated hyperactivity of T and B lymphocytes in SLE [131]. In this study, the basal expressions of p38 MAPK in CD4^+^ T lymphocytes, CD8^+^ T lymphocytes, and B lymphocytes had been shown to be significantly higher in SLE patients, and the expression of phospho-p38 MAPK in CD4^+^ T lymphocytes, CD8^+^ T lymphocytes, and B lymphocytes, and phospho-JNK in CD8^+^ T lymphocytes and B lymphocytes was also significantly elevated in SLE patients upon the activation by IL-18, exhibiting significant correlation with the plasma concentrations of Th1 chemokine CXCL10. Furthermore, the expression of phospho-JNK in IL-18-activated CD8^+^ T lymphocytes and the relative percentage (%) fold increase of the expression of phospho-JNK upon IL-18 activation in B lymphocytes were significantly correlated with SLE disease activity index. Therefore, the inflammation-mediated activation of JNK and p38 MAPK signaling pathways in T and B lymphocytes can be the underlying intracellular mechanism causing lymphocyte hyperactivity in SLE.

## 8. Pattern-Recognition Receptors in SLE: Friend or Foe

An infectious etiology of SLE has been a longstanding hypothesis [132–134] and with the discovery of PRRs in SLE, the role of bacteria and viruses in the pathogenesis of SLE has been invigorated. PRRs can alert and activate the innate immune system through recognizing the conserved molecular patterns to distinguish extrinsic pathogen-associated molecular patterns (PAMPs) and endogenous danger-associated molecular patterns (DAMPs). Several PRRs participated in the recognition of viral components, such as genomic DNA and RNA, in a replication-independent way. Additionally, cells express intracellular RNA helicases that function as PRRs of actively replicating viruses [135]. These PRRs are also essential in establishing antiviral immunity by triggering type I interferon responses.

### 8.1. Toll-Like Receptor in SLE

Being the most studied PRRs, Toll-like receptors- (TLRs-) mediated intracellular signaling is a crucial link between innate and adaptive immunity [136], which principally sense structurally conserved molecular motifs called PAMPs for triggering NF-*κ*B, p38 MAPK, JNK, and the IFN pathways, which results in the translocation of transcription factors, cytokine modulation, and IFN-stimulated gene regulation leading to inflammatory responses [137]. The stimulation of TLR by PAMPs is an important prerequisite for the induction of various autoimmune diseases [138]. To date, at least 10 human TLRs have been identified, and the functions of human TLR1-9 have been characterized [138, 139]. Cell surface TLRs (TLR-1, 2, 4, 5, and 6) are designed for the engagement of extracellular pathogens, whereas the intracellular TLRs (TLR-3, 7, 8, and 9) are against intracellular pathogen-derived products [140].

 Animal studies of SLE have indicated that TLRs are important in the pathogenesis of lupus mouse. For instance, in myeloid differentiation primary response gene (MyD) 88-deficient MRL/MpJ-Fas(lpr) (MRL/lpr) mice, both MyD88-dependent and -independent innate signals were found to play a crucial role in the development of autoimmune nephritis [141]. Treatment of lupus-prone mice with a dual inhibitor of TLR-7 and TLR-9 was found to lead to the reduction of autoantibody production and amelioration of disease symptoms [142]. Lupus-prone mice deficient in TLR-7 also failed to generate antibodies against RNA-containing antigens such as Smith, which decreased lymphocyte activation and serum IgG [143]. Conversely, the absence of TLR-9 can exacerbate the disease activity by the activation of lymphocytes and plasmacytoid dendritic cells (pDCs), inducing the subsequent increase of serum IgG and IFN-*γ* [143]. Emerging evidence revealed that TLR-9 was involved in class-switching to pathogenic autoantibody production in SLE [144, 145]. Accordingly, patients with active SLE had been shown to have upregulated expression of TLR-9 in peripheral blood memory and plasma B lymphocytes, suggesting that endogenous nucleic acids released during apoptosis may stimulate B lymphocytes via TLR-9 and contribute to SLE pathogenesis [146]. Upregulated expression of TLR-7 and TLR-9 mRNA, together with IFN-*γ* mRNA in PBMC, may also contribute to the pathogenesis of human lupus [147]. Consistently, other study also revealed that PBMCs of SLE patients with a higher expression of TLRs are more prone to be activated by diverse TLR ligands when compared to HCs [147, 148], suggesting that the innate immune response for extracellular pathogens and self-originated DNA plays immunopathological roles via TLR activation in SLE.

 Recent study by our group found that antagonist-mediated diminished intracellular TLRs might act as potent activators of innate immune responses involved in the higher prevalence of human papillomavirus infection (HPV) in SLE [149]. TLR antagonist, such as hydroxychloroquine, might decrease the expression of intracellular TLRs in SLE patients, thereby increasing the risk of acquiring HPV infection. Moreover, high-risk HPV infections may play a predominant role in further downregulating the expression of intracellular TLR in SLE patients with HPV infection resulting in a higher prevalence of persistent infection, suggesting that the avoidance of stimulation and downregulation of the innate immune system, which might permit persistence of HPV in SLE, is evidently part of an immune evasion strategy used by oncogenic HPV establishing of persistence infection [149].

### 8.2. Nucleotide-Binding Oligomerization Domain Containing 2 in SLE

In contrast to the well-elucidated membrane-bound TLRs, cytoplasmic nucleotide binding oligomerisation domain (NOD) receptors are a new family of PRRs for the recognition of extracellular PAMPs [150, 151]. Two NOD-like receptor (NLR) proteins, namely, NOD1 and NOD2, can participate in the signaling events triggered by host recognition of specific motifs of bacterial peptidoglycans (PGNs) and, upon activation, induce the production of proinflammatory mediators [150]. NOD1 recognizes products from gram-negative bacteria (diaminopimelic acids), whereas NOD2 senses muramyl dipeptide (MDP), a peptidoglycan derived peptide from gram-negative as well as gram-positive bacteria [152]. It has been shown that NLRs complement and synergize with TLRs in innate immune responses [153–156]. NLRs are associated with Crohn's disease and inflammatory arthritis [155–157]. However, the precise mechanisms by which NOD-mediated recognition of PGNs in the pathogenesis of inflammatory diseases are still unclear. Apart from the putative link between the genetic variants of NOD2 and SLE [158–163], little is known about the expression and function of NOD2 in SLE [164]. Our recent study demonstrated an overexpression of NOD2 in monocyte of immunosuppressant naïve SLE patients with longer process might lead to activation of PBMCs to produce proinflammatory cytokines, implicating the innate immune response for extracellular pathogens in immunopathological mechanisms in SLE [165]. Conversely, immunosuppressive therapy may downregulate the expression of NOD2 in CD8^+^ T, monocytes, mDCs, and pDCs in SLE which subsequently reduce regulatory cytokine IL-10, allowing for an aberrant inflammatory response contributing towards the regulation of immunopathological mechanisms of SLE, at the expense of increasing risk of bacterial infection [165]. NOD1 expression in PBMC subsets of SLE patients and HCs could not be detected using flow cytometry [165].

 Recently, increased prevalence of HPV and tuberculosis (TB) in SLE has been reported by our group and others [166–169]. Whether the immune evasion strategy, specific bacteria, or virus could escape PRRs recognition, establishment of persistent infection in SLE playing a significant part in host-pathogenic interaction need further considerations. Further elucidation of the infectious process and immune response against infections and exploration of the efficacy of agonists as therapeutic tools for eliminating infected cells in SLE will be worth investigation.

## 9. Conclusion

The understanding of the immunopathologic mechanisms of SLE has been gradually evolving with budding studies on assessing the activation of monocytes, T, and B lymphocytes upon stimulation of various stimuli and also underlying intracellular signaling mechanisms. This further enhanced our current and limited knowledge regarding the cellular mechanism and pathway in the immunopathogenesis of SLE, which had shed light on developing potential and novel therapies in treating this chronic immunological disorder. Therapeutic inhibitors of the pathways of JNK or p38MAPK [170, 171] and antibodies against IL-21, CXCL13 [172, 173], and TLR [174, 175] have been shown to exhibit some promising beneficial effects. Hopefully, with the advent of more advanced technology and emergence of more studies, our understanding for this elusive disease can be further strengthened in the future.
